# VIRdb 2.0: Interactive analysis of comorbidity conditions associated with vitiligo pathogenesis using co-expression network-based approach

**DOI:** 10.12688/f1000research.25713.2

**Published:** 2021-02-01

**Authors:** Priyansh Srivastava, Mehak Talwar, Aishwarya Yadav, Alakto Choudhary, Sabyasachi Mohanty, Samuel Bharti, Priyanka Narad, Abhishek Sengupta

**Affiliations:** 1 Amity Institute of Biotechnology, Amity University, Noida, Uttar Pradesh, 201301, India

**Keywords:** Vitiligo, Database, Comorbidity Network, Differential Genes, co-expressed Genes

## Abstract

Vitiligo is a disease of mysterious origins in the context of its occurrence and pathogenesis. The autoinflammatory theory is perhaps the most widely accepted theory that discusses the occurrence of Vitiligo. The theory elaborates the clinical association of vitiligo with autoimmune disorders such as Psoriasis, Multiple Sclerosis and Rheumatoid Arthritis and Diabetes. In the present work, we discuss the comprehensive set of differentially co-expressed genes involved in the crosstalk events between Vitiligo and associated autoimmune disorders (Psoriasis, Multiple Sclerosis and Rheumatoid Arthritis). We progress our previous tool, Vitiligo Information Resource (VIRdb), and incorporate into it a compendium of Vitiligo-related multi-omics datasets and present it as VIRdb 2.0. It is available as a web-resource consisting of statistically sound and manually curated information. VIRdb 2.0 is an integrative database as its datasets are connected to KEGG, STRING, GeneCards, SwissProt, NPASS. Through the present study, we communicate the major updates and expansions in the VIRdb and deliver the new version as VIRdb 2.0. VIRdb 2.0 offers the maximum user interactivity along with ease of navigation. We envision that VIRdb 2.0 will be pertinent for the researchers and clinicians engaged in drug development for vitiligo.

## Introduction

Vitiligo is a long-term pigmentary disorder of ambiguous origins. It is a multifactorial disease described by a plethora of theories ^1^. As stated in neural theory, the dysfunction of the sympathetic nervous system affects melatonin production which leads to depigmentation ^2^. The oxidative stress hypothesis states that vitiliginous skin is the result of the overproduction of reactive oxygen species such as H _2_O _2_ leading to the demolition of melanocytes that results in depigmentation ^3^. The zinc-α2-glycoprotein (ZAG) deficiency hypothesis exploits the integral role of ZAG, which acts as a keratinocyte derived factor responsible for rapid melanocyte production ^4^. Absence of ZAG results in the detachment of melanocytes from epidermis which leads to vitiligo ^4^. Chronic hepatitis C virus and autoimmune hepatitis have strong union with vitiligo and account for viral origins ^5^. According to Intrinsic theory, melanocytes may have natural defects such as abnormal rough endoplasmic reticulum or deficiency of growth factors which could lead to melanocyte apoptosis ^6^. The Autoinflammatory theory is the most widely accepted causation supported by strong evidence ^7^. The hypothesis is mainly based on the clinical association of vitiligo with several other autoimmune disorders like psoriasis (PS), multiple sclerosis (MS) or rheumatoid arthritis (RA) ^8– 11^.

Vitiligo Information Resource (VIRdb) is home to statistically significant and manually curated information about vitiligo. In our previous work, we integrated drug-target and systems-based approaches to generate a comprehensive resource for vitiligo-omics. Vitiligo Information Resource (VIRdb) that integrates both the drug-target and systems approach to produce a comprehensive repository entirely devoted to vitiligo, along with curated information at both protein level and gene level along with potential therapeutics leads. These 25,041 natural compounds are curated from Natural Product Activity and Species Source Database. VIRdb is an attempt to accelerate the drug discovery process and laboratory trials for vitiligo through the computationally derived potential drugs. It is an exhaustive resource consisting of 129 differentially expressed genes, which are validated through gene ontology and pathway enrichment analysis. We also report 22 genes through enrichment analysis which are involved in the regulation of epithelial cell differentiation. At the protein level, 40 curated protein target molecules along with their natural hits that are derived through virtual screening ^12^. Data stored in VIRdb is linked with major public databases making it a cross-functional database ^12– 17^. It is an encyclopedic resource consisting of 129 differentially expressed genes (DEGs) in different phenotypes of vitiligo ^12^. It also holds 40 curated protein targets along with their natural ligands that are derived through virtual screening ^12^. In the present work, we aim to provide a comprehensive set of DEGs involved in the crosstalk events of vitiligo and associated autoimmune disorders (PS, MS, RA). We further investigated the interactions of the gene through comorbidity gene-gene interaction network (GGI). All data have been synthesized using standardized differential-expression pipelines. The data presented in the present study have been integrated on the VIRdb along with the previously published datasets. The paper also discusses the major updates and expansions in the VIRdb and delivers the new version as VIRdb 2.0 ^18^. The specific goals of the study is to offer an engaging user-interface along with interactive visualizations for a comprehensive understanding of the disease pathogenesis. Data descriptors have been added to the browsing interface for effortless navigation through the data.

## Results

### Differentially expressed genes

 The expression set for each disease (vitiligo, MA, RA and PS) constituted expression values of 27,338 probe IDs that belong to the Affymetrix GPL570 platform. After differential expression analysis, 834 differential genes were expressed in vitiligo (Lesional, Peri-lesional and Non-lesional together) out of which 639 genes were over-expressed and 195 genes were under-expressed ( *Extended data*, Appendix-I). In RA samples a total of 938 genes were expressed, out of which 422 genes were over-expressed and 516 genes were under-expressed in the diseased condition ( *Extended data*, Appendix-I). For MS 1783 differentially expressed genes were filtered in which 710 genes were over-expressed and 1073 genes were under-expressed ( *Extended data*, Appendix-I). In PS, 4016 differentially expressed genes were filtered out in which 2088 were over-expressed and 1928 were under-expressed ( *Extended data*, Appendix-I).

### Correlated Expressions

 Pearson’s correlation analysis on DEGs (vitiligo) produced 397 pairs that were positively correlated with each other ( *Extended data*, Appendix-II ^19^). Interestingly, we found none of the gene-pairs to be under-expressed in vitiligo except the *FKBP5-CUL7*. In PS there were a total of 1089 positively correlated pairs out of which the 45 pairs were showing under-expression and 1044 pairs were showing over-expression ( *Extended data*, Appendix-II ^19^). A skewness towards over-expressed pairs can be seen in the pairs positively correlated with PS. Pearson’s correlation testing of MS-DEGs generated 767 positively correlated pairs, with 623 under-expressed pairs and 144 over-expressed pairs ( *Extended data*, Appendix-II ^19^). Testing of RA-DEGs produced the least number of positively correlated pairs, i.e. 411, in which 301 pairs were under-expressed and 110 pairs were over-expressed ( *Extended data*, Appendix-II ^19^). It showed skewness towards the under-expressed pairs.

### Intersecting genes

**Figure 1.**
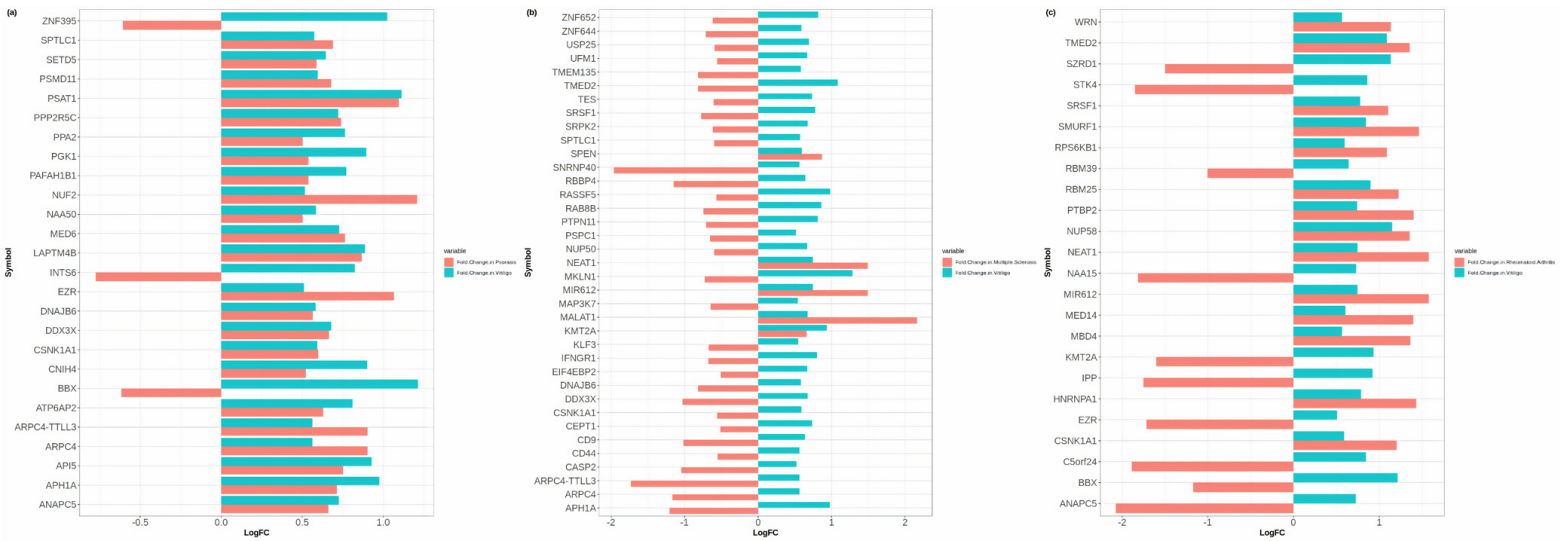
Intersecting genes with relative LogFC. ( **a**) Positively correlated DEGs common between vitiligo and PS; ( **b**) Positively correlated DEGs common between vitiligo and MS; ( **c**) Positively correlated DEGs common between vitiligo and RA.

 PS shared 26 differentially expressed (positive co-expressed) genes with vitiligo. In total, 23 differentially expressed genes showed similar expression (Over-expression) both in the vitiligo and PS ( Figure 1a). However, *ZNF395*, *INTS6* and *BBX* showed an opposite expression in PS ( Figure 1a). MS and vitiligo shared 37 differentially expressed (positive co-expressed) genes, all of which showed contradictory expressions except *SPEN*, *NEAT1*, *MIR612*, *MALT1* and *KMT2A* (similar expression) ( Figure 1b). RA shared 24 differentially expressed (positive co-expressed) genes with the vitiligo and showed randomness in the expression ( Figure 1c).

### Network Attributes

**Figure 2.**
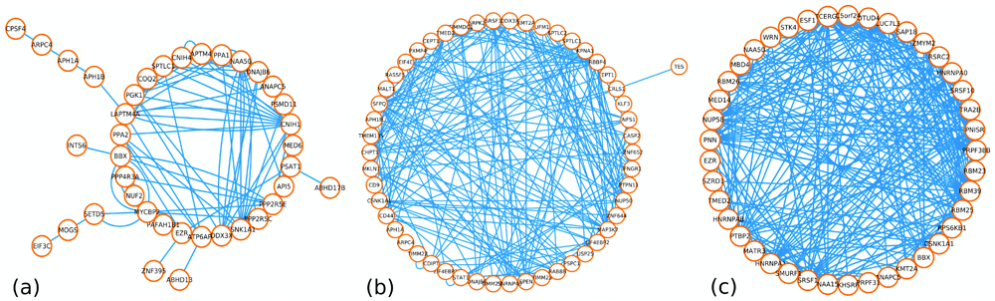
Gene-gene interaction networks designed using GeneMania. ( **a**) GGI Network for common positively correlated DEGs between vitiligo and PS. ( **b**) GGI Network for common positively correlated DEGs between vitiligo and MS. ( **c**) GGI Network for common positively correlated DEGs between vitiligo and RA.

 The GGI network of the intersecting positively correlated DEGs of vitiligo and PS holds 38 nodes with 93 edges denoting the co-expression, physical interactions and pathway relationships mined from literature ( *Extended data*, Appendix-IV ^19^) ( Figure 2a). GeneMania’s algorithm also fetched 12 intermediary genes to connect the shared 26 differentially expressed (positive co-expressed) genes ( *Extended data*, Appendix-IV ^19^). the network for vitiligo and MS holds 51 nodes and 219 edges mined from literature, having 14 intermediary genes ( *Extended data*, Appendix-IV ^19^) ( Figure 2b). The GGI network of RA and vitiligo constituted 33 nodes and 470 edges, out of which 9 nodes (genes) were intermediary genes that were fetched by GeneMania’s algorithm ( *Extended data*, Appendix-IV ^19^) ( Figure 2c).

### Enrichments and annotations

**Figure 3.**
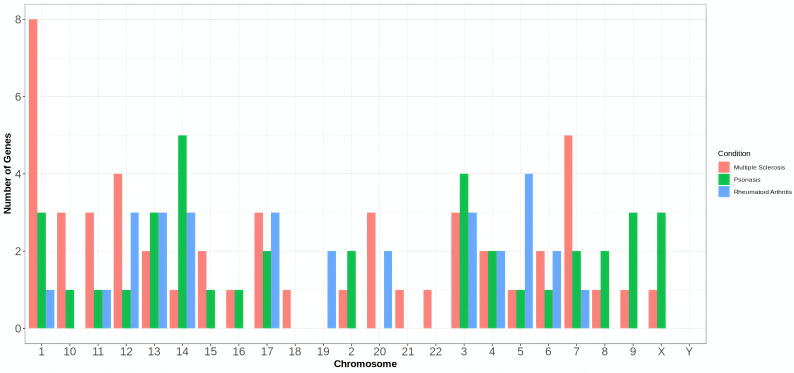
Chromosomal distribution of the intersecting DEGs. Red bars show the distribution of the intersecting DEGs between vitiligo and MS. Green bars show the distribution of the intersecting DEGs between vitiligo and PS. Blue bars show the distribution of the intersecting DEGs between vitiligo and RA.

 Mechanical annotation of the genes from the GGI network of vitiligo and PS shows the highest expressivity in chromosome 14. Chromosome 18-21 doesn’t express at all ( *Extended data*, Appendix-V ^19^) ( Figure 3). Genes of the GGI network of vitiligo and MS were expressed mainly on chromosomes 1,7 and 12 ( *Extended data*, Appendix-V ^19^) ( Figure 3). Nodes from the GGI network of vitiligo and RA showed no expression through chromosomes 2, 8-10, 15, 16, 18, 21 and 22 ( *Extended data*, Appendix-V ^19^) ( Figure 3). Expression was not seen through chromosome Y in any case ( *Extended data*, Appendix-V ^19^) ( Figure 3). Intersecting genes of the GGI network of vitiligo and PS were mostly (23/36) enrichment with “Cellular protein metabolic process” as a biological process ( *Extended data*, Appendix-VI ^19^). MS and vitiligo network was enriched in “Regulation of innate immune response” (8/50) as a biological process ( *Extended data*, Appendix-VI ^19^). “RNA binding” (24/32) was enriched as the biological process for the nodes of the RA and vitiligo network ( *Extended data*, Appendix-VI ^19^).

### Updates and expansions

**Figure 4.**
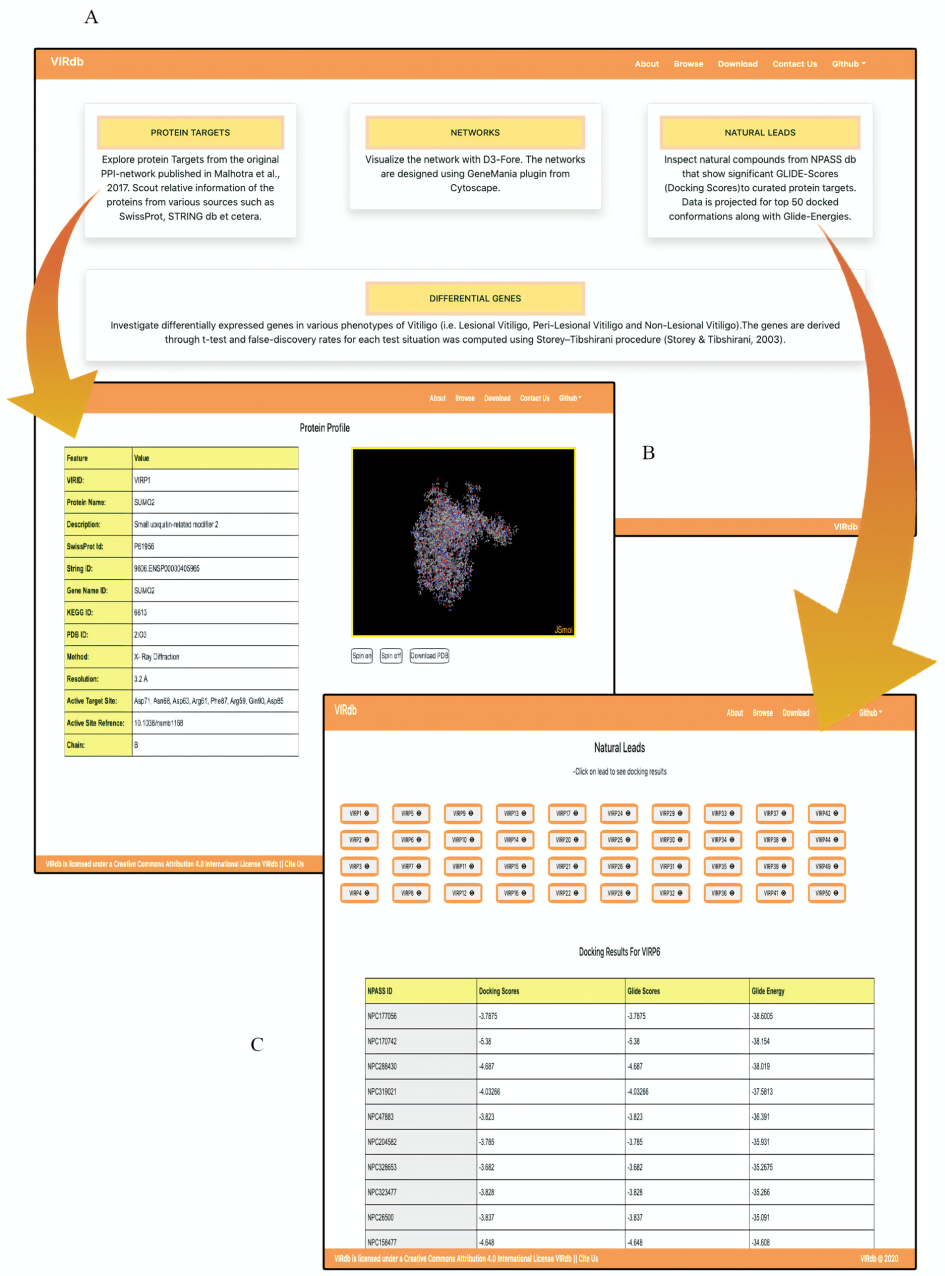
Improvisations on the User-interface of the VIRdb. ( **a**) The browsing section has been redesigned with descriptors for easy navigation through the database. ( **b**) JSmol has been optimized for various visualization styles which can be enabled using right-click. ( **c**) The Natural lead section has been reduced to the top entries based on the significant Glide-Scores.

**Figure 5.**
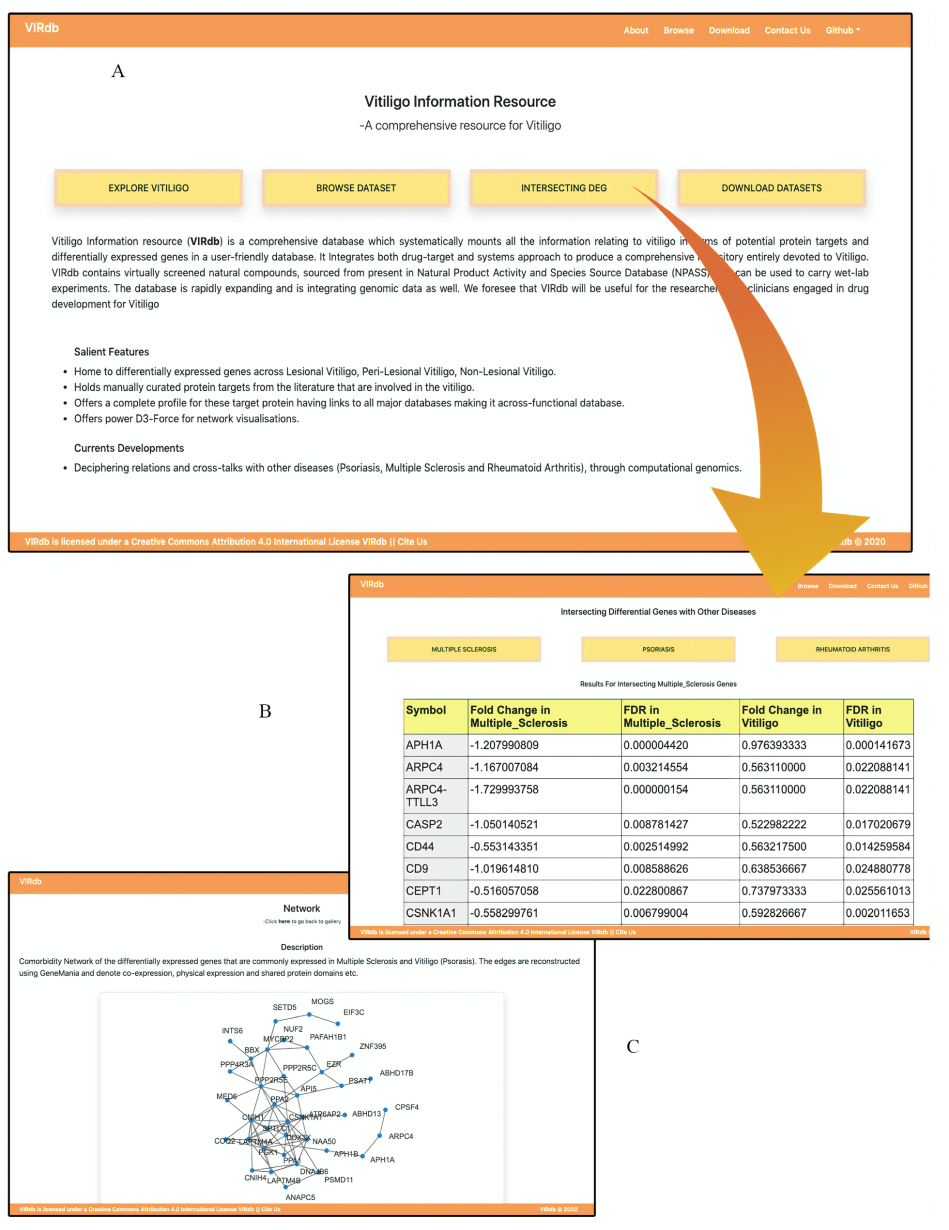
( **a**) Revamped home page of the VIRdb 2.0 with all the section tabs for easier navigation; ( **b**) Intersection section displays the positively co-expressed DEGs across PS, vitiligo, MS and RA. Gene symbols are cross-connected with GeneCards db. ( **c**) Network gallery section with D3-force layout. It offers a highly interactive network visualization of the comorbidity networks.

VIRdb 2.0 ^18^ offers an engaging user-interface along with interactive visualizations. Data descriptors have been added to the browsing interface for effortless navigation through the data ( Figure 4a) ^12^. The JSmol visualizer of the protein profiles has been optimised for quick visualizations ( Figure 4b) ^12^. The profiles have cross-connectivity to other databases through their respective Accession IDs. The user can now visualize the protein structure in various styles (i.e. cartoon, ribbons, etc.) using JSmol options. The downloadable structure has been already prepared for molecular docking procedures (i.e. removed the water, ions, chargers, ligands and minimized with OPLS 2005) ( Figure 4b) ^12^. The natural leads section has been reduced to the top 50 computational hits against the protein targets ( Figure 4c) ^12^. The user can browse through fewer compounds before setting up wet lab experimentations ( Figure 4c) ^12^. Intersecting positively co-expressed DEGs between vitiligo and other conditions (PS, RA and MS) is the new addition to the database ( Figure 5b). The section consists of four columns which are connected to GeneCards via gene symbols. The section holds expression status (Overexpression/Underexpression) of the positively co-expressed DEGs between vitiligo and associated conditions ( Figure 5b). The GGI networks which are made using the shared positively co-expressed DEGs between vitiligo and other conditions can be viewed in the network gallery section ( Figure 5c). The networks are highly interactive and can be simulated with mouse clicks.

**Figure 6.**
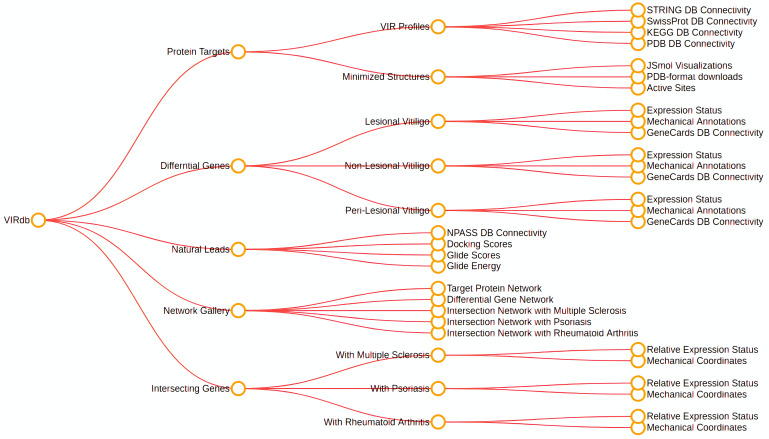
Updated Database Schema for Vitiligo Information Resource 2.0. Network Gallery and Intersecting Gene sections have been added with cross-connectivity and D3 force-directed graph visuals.

## Methods

### Data Curation

 Raw datasets from the microarray studies for RA (GSE56649), PS (GSE14905), MS (GSE21942), and vitiligo (GSE65127) were downloaded from the Gene Expression Omnibus (GEO) ^20^. To maintain the uniformity of the downstream analysis, experiments with Affymetrix GPL570 platform were taken into consideration. Expression values were extracted from the raw CEL files using the affy (version 1.66.0) library in R version 3.6 ^21^. Expression datasets were normalized using the robust multichip averaging method for background corrections ^22^. After the normalization, the IQR method was used with a standard cut-off of 0.5 to remove the low expression values from the datasets using genefilter (version 1.70.0) library ^23^. Each dataset was divided into two groups based on the phenotype of the samples, i.e. “Experiment” (disease phenotypes) and “Control” (healthy phenotypes). Specifically, for vitiligo expression set, “Experiment” groups constituted of lesional, peri-lesional and non-lesional vitiligo samples together. Scripts used for computational analysis of data generated through microarray experiments are available on Github: https://github.com/pnarad/Micro-Array-Data-Analysis ^24^.

### Differential Expression Analysis

 Standard linear models’ library limma (version 3.44.3) was used to perform the differential expression analysis ^25^. The t-test was performed with each gene expression value to examine variations across groups (“Experiment” vs “Control”). Benjamini Hochberg’s false discovery rate was computed to filter the significant multiple testings ^26^. P-value cut-off of 0.05 and adjusted-P-value (FDR) cut-off of 0.03 was used to filter the significant test results. Log of fold change (LogFC) across groups was calculated alongside with standard “limma” functions to explore the expression status of the differentially expressed probes ids. Probe Ids with LogFC < -0.5 were annotated as underexpressed and those with LogFC > 0.5 were annotated as overexpressed in “Experiment” samples and the remaining probe ids were dropped (i.e. -0.5 < LogFC < 0.5). 

### Probe mappings

 The respective feature dataset that contains the gene symbol mapping for the probe id fetched from the GEO using “GEOquery” ^27^. The differentially expressed probe ids were mapped to their respective gene symbols programmatically ^28^. The gene symbols that were mapped to multiple probe ids with different expression status (i.e. ambiguity in over/under-expression) were removed using dplyr (version 1.0.1) methods ^29^. The gene symbols which were mapped on multiple probe ids with uniform expression (i.e. uniformity in over/under-expression) were averaged over the calculated parameters (i.e. P-value, adjusted P-value, average expression etc.). The final datasets constituted unique differentially expressed genes in “Experimental” samples of all the four diseases.

### Correlations and intersections

 The correlated pairs (similar expressions across the samples) were formed within the disease groups (i.e. MS, RA, PS and vitiligo) individually. Initially, the differentially expressed gene symbols were reverse mapped to their respective probe ids for extraction of expression values. Pearson’s correlation test was performed to compute the correlation coefficient. The correlation matrix was filtered for correlations having a correlation coefficient > ±0.9. The probe IDs were re-mapped to the gene symbols along with LogFC. The negatively correlated pairs were removed using the LogFC (i.e. different expression status of gene in a pair), and only positive correlated differential pairs were taken for further analysis ^30^. Cytoscape (version 3.8) was used to create four individual co-expression networks using the differentially expressed and positively correlated gene pairs ^31^. The co-expression networks for MS, RA and PS were individually intersected with the co-expression network of vitiligo for evaluation of commonly expressed genes across diseases.

### Gene-gene interaction network reconstruction

 The intersecting genes were fed to GeneMania for edge-reconstruction of the comorbidity gene interaction networks (i.e. vitiligo with RA, vitiligo with MS and vitiligo with PS) ^32^. The intersecting gene symbols were joined using annotated edges of GeneMania algorithm. We dropped “predicted” edges and selected the rest of the edges which were sourced from the literature. The networks were examined using Cytoscape and the largest connected subnetworks were isolated.

### Annotations and ontologies

The gene symbols from each dataset were fed to the g:Profiler server for gene ontologies ^33^. The Benjamini–Hochberg false discovery rate of 0.03 was chosen with an annotated domain scope for enrichment analysis. Finally, network nodes were annotated using the biomaRt (version 2.44.1) data mining tool for chromosome number, start base-pair and stop base-pairs ^34^. The three comorbidity network datasets were mapped with their respective LogFC for meaningful insights

### Updated architecture and design

The previous schema of the database was updated with the new addition of Network Gallery and Intersecting Gene’s sections ( Figure 3). Bootstrap objects were added for styling along with HTML 5 and CSS in the front-end stack. JavaScript was added for dynamic visualizations of JSmol viewer and D3 force-directed networks based on Verlet integration ^35– 37^.

### Operation

 The VIRdb 2.0 ^18^ is available online as an open-source database. The user can access the database through any platform (Linux, Mac OS, or Windows). There are no specific system requirements to browse the database. The user can browse through different sections using the easy to use interface.

### Implementation

The database is implemented in various sections. Intersection section displays the positively co-expressed DEGs across PS, vitiligo, MS, and RA. Gene symbols are cross connected with GeneCards db. Network gallery section of the database offers a highly interactive network visualization of the comorbidity networks. The JSmol viewer has been optimized for various visualization styles which can be enabled using right-click. The user can visualize the structure in various styles such as cartoons, ribbons etc. The Natural lead section has been reduced to the top entries based on the significant Glide-Scores.

## Discussion

 The present study discusses the comorbidities of the vitiligo with known associative disorders based on auto-inflammatory theory ^8– 11^. With the aid of statistical procedures, we found significant DEGs that show correlated expressions in vitiligo, PS, MS and RA. *ZNF395* which activates a subset of ISGs including the chemokines *CXCL10* and *CXCL11* in keratinocytes was co-expressed between the PS and vitiligo ^38^. *CXCL10* and *CXCL11*, are expressed in skin keratinocytes and are involved in the development of proinflammatory skin diseases such as vitiligo ^38^. *ZNF395* was over-expressed in our dataset of positively co-expressed DEGs from vitiligo samples and was under-expressed in samples of PS ( *Extended data*, Appendix-III ^19^). *ZNF395* has direct associations with Huntington’s disease and might be a crucial biomarker in vitiligo-PS auto-immune progression as well ^39^. *CSNK1A1*, which was expressed in all the conditions showed under-expression in MS and therefore could be used as a biomarker for MS ( *Extended data*, Appendix-III ^19^). It is also interesting to see that most of the shared DEGs in PS and RA show uniform expression like vitiligo. However, the expressions of DEGs shared with MS show contradictory expressions, which points towards a weaker association between vitiligo and MS. The presented comorbidity networks hold many new DEGs which are shared among the auto-immune diseases. These DEGs can be explored for their cross-talks events as supported by auto-inflammatory theory ^7^.

VIRdb 2.0 ^18^ integrates statistically significant DEGs that could be responsible for crosstalk events between vitiligo, PS, MS and RA. VIRdb 2.0 incorporates all the attributes of the previous version and projects them in a more user-friendly database. The intersecting DEGs with other diseases (PS, MS and RA) are projected with their expression status in diseases. VIRdb 2.0 also utilizes a network approach to project positively co-expressed genetic interaction networks of the intersecting differentially expressed genes. VIRdb 2.0 also offers the maximum user interactivity with the D3 layouts. The users can now visualize the GGI networks and minimized protein structs directly on the database. The visualization tools (JSmol and D3-simulator) allow switching between various poses. The datasets can be downloaded in a zipped archive with few clicks from the download section. Thus, VIRdb 2.0 will be pertinent for the researchers and clinicians engaged in drug development and genomics of vitiligo.

## Conclusion

 We present VIRdb 2.0, intersecting differential networks that could be responsible for cross-talks events between vitiligo, PS, MS and RA. The presented networks are designed using the common positively co-expressed DEGs in the disease. The VIRdb 2.0 inherits all the previous datasets of VIRdb and incorporates new datasets that are discussed in the present study. Future versions will include data submission capabilities and functional-omics perspectives.

## Data availability

### Underlying data

### Extended data

Figshare: VIRdb 2.0: Interactive analysis of comorbidity conditions associated with vitiligo pathogenesis using co-expression network-based approach. https://doi.org/10.6084/m9.figshare.12776468.v1 ^19^.

This project contains the following extended data:

- Appendix-I (Differential Genes).xlsx. (Results of the differential expression analysis.)
- Appendix-II (Positive Correlations).xlsx. (Results of the Pearson’s correlation testing.)
- Appendix-III: (Intersection results of the differential genes.)
- Appendix-IV (Networks).xlsx. (Network files of the GGI networks constructed through GeneMania.)
- Appendix-V (Annotations).xlsx. (Results of the gene annotation using the BioMart Data Mining tool.)
- Appendix-VI (Ontologies).xlsx. (Results of Gene Ontology enrichment analysis.)

**Scripts used for data analysis are available from:** https://github.com/pnarad/Micro-Array-Data-Analysis.

**Archived scripts at time of publication:** https://doi.org/10.5281/zenodo.3975638 ^24^.

**License:** MIT License.

## Software availability

**VIRdb 2.0 is available at:** https://vitiligoinfores.com/.

**Source code available from:** https://github.com/pnarad/VIRdb.

**Archived source code at the time of publication:** https://doi.org/10.5281/zenodo.3975634 ^18^.

**VIRdb license:** Creative Commons Attribution 4.0 International.

**Source code license:** Creative Commons Zero “No rights reserved” data waiver.
