# Supplementary material for: VIRdb 2.0: Interactive analysis of comorbidity conditions associated with vitiligo pathogenesis using co-expression network-based approach
Source: F1000Res. 2021 Feb 1;9:1055. Originally published 2020 Aug 27. [Version 2] doi: 10.12688/f1000research.25713.2 (PMC7953917; doi:10.12688/f1000research.25713.2)
Supplement: Peer review history

# Peer review: Reviewer response for version 2

**DOI:** 10.5256/f1000research.54266.r78583

**Published:** 2021-03-11

**Authors:** Dinesh Gupta

**Affiliations:** 1 Translational Bioinformatics Group, International Centre for Genetic Engineering and Biotechnology (ICGEB), New Delhi, Delhi, India

My review comments have been adequately addressed by the authors. I recommend the revised manuscript for indexing.

Are the conclusions about the tool and its performance adequately supported by the findings presented in the article?

Yes

Is the rationale for developing the new software tool clearly explained?

Yes

Is the description of the software tool technically sound?

Yes

Are sufficient details of the code, methods and analysis (if applicable) provided to allow replication of the software development and its use by others?

Partly

Is sufficient information provided to allow interpretation of the expected output datasets and any results generated using the tool?

Partly

Reviewer Expertise:

Translational Bioinformatics, Systems biology, database, and algorithm development.

I confirm that I have read this submission and believe that I have an appropriate level of expertise to confirm that it is of an acceptable scientific standard.

# Peer review: Reviewer response for version 1

**DOI:** 10.5256/f1000research.28377.r75274

**Published:** 2021-01-11

**Authors:** Animesh A Sinha

**Affiliations:** 1 Department of Dermatology, University at Buffalo, The State University of New York, Buffalo, NY, USA

This is an overall well written paper comparing transcriptional datasets in vitiligo to other autoimmune disease datasets.

 Building on previous work, the authors further develop their software for integrative analysis.

**Comments:**

1. Intro: the Specific goals of the study and the previous work by the authors relevant to this study should be further explained.
2. It is not clear that all published and available datasets for VL and the other autoimmune diseases have been incorporated into the program.  A Table of all relevant studies should be provided in the manuscript or supplemental data.
3. The site itself would benefit from tutorials and a better search function.
4. Some more extensive data on disease intersecting genes and functional pathways should be included.

Are the conclusions about the tool and its performance adequately supported by the findings presented in the article?

Partly

Is the rationale for developing the new software tool clearly explained?

Yes

Is the description of the software tool technically sound?

Yes

Are sufficient details of the code, methods and analysis (if applicable) provided to allow replication of the software development and its use by others?

Partly

Is sufficient information provided to allow interpretation of the expected output datasets and any results generated using the tool?

Partly

Reviewer Expertise:

Immunogentics

I confirm that I have read this submission and believe that I have an appropriate level of expertise to confirm that it is of an acceptable scientific standard, however I have significant reservations, as outlined above.

# Response

**Published:** 2021-01-27

**Authors:** Abhishek Sengupta

**Affiliations:** Amity University, India

Dear Sir,

 We thank you for the generous and concise comments on our manuscript. We have incorporated the suggestions and changes have been added to the manuscript text also.

 Q.1 Intro: the Specific goals of the study and the previous work by the authors relevant to this study should be further explained.

**As per the suggestion, we have incorporated a few lines about the specific goals of the study as well as the explained our previous work briefly. We have given the reference to our previously published paper to be explored in more detail.**

 Q.2 It is not clear that all published and available datasets for VL and the other autoimmune diseases have been incorporated into the program.  A Table of all relevant studies should be provided in the manuscript or supplemental data.

**We have mentioned in our previous paper the dataset that was used for Vitiligo and the inclusion and exclusion criterion for all the available datasets. To briefly answer your question, Gene Expression Omnibus was manually queried using “(“vitiligo” (MeSH Terms) OR vitiligo (All Fields)) AND (“humans” (MeSH Terms) OR “Homo sapiens” (Organism) OR homo sapiens (All Fields))”. The returned results were manually curated for microarray experiments having healthy controls against affected individuals only for both the VL and other autoimmune diseases that we have considered in our work. As no plausible Our primary focus was on microarray data and no plausible data was available having an adequate amount of data for analysis so other autoimmune diseases were excluded. We aim to work on more data and include more information in the further prospects of this project.**

 Q.3 The site itself would benefit from tutorials and a better search function.

**A short tutorial has been incorporated at the Home page highlighting the sections and the process of retrieval from the sections. Complete code for accessing the database is already available at GitHub. A new page has been created to highlight "what's new in this release".**

 Q.4 Some more extensive data on disease intersecting genes and functional pathways should be included.

**A network gallery is available under the “Browse Section”. The visualization of the network of the intersecting genes with D3-Fore is available. These networks have been generated using GeneMania plugin from Cytoscape. Further, the analysis was also done to elaborate the Gene Ontology for the intersecting DEGs, the data is available in Appendix-VI. Further, we aim to build more towards the on this in our future studies. ADMET studies have also been included.**

# Peer review: Reviewer response for version 1

**DOI:** 10.5256/f1000research.28377.r75389

**Published:** 2021-01-04

**Authors:** Rasheedunnisa Begum, Mitesh Dwivedi

**Affiliations:** 1 C.G. Bhakta Institute of Biotechnology, Faculty of Science, Uka Tarsadia University, Surat, Gujarat, India; 2 Department of Biochemistry, Faculty of Science, Maharaja Sayajirao University of Baroda, Vadodara, Gujurat, India

The study presented by Srivastava *et al.* (2020) is a unique approach to elucidate the link between Vitiligo and associated autoimmune disorders through a set of differentially co-expressed genes involved in their pathogenesis. For this, authors have designed a valuable tool called VIRdb 2.0: an integrative database that can be utilized by researchers and clinicians for drug development for vitiligo.

 Authors have well explained the need and rationale for developing this new software tool. This database has been connected to KEGG, STRING, GeneCards, SwissProt, & NPASS. The different datasets can be easily downloaded by the user such as protein target information, differential expression of genes, docking results with natural compounds, and interaction network. In addition, VIRdb 2.0 also has a separate blog section where researchers can write interesting and updated informative blogs.

 Overall, the software seems to be user friendly, though certain information and functionalities about submission of data by other researchers working in the fields of Vitiligo and its associated diseases is lacking in this version. The below are a few suggestions and concerns which authors should consider and implement in the future version of the VIRdb 2.0.

**Conceptual concerns:**

1. Though VIRdb 2.0 has included information on certain autoimmune disorders associated with Vitiligo (i.e. Psoriasis, Multiple Sclerosis and Rheumatoid Arthritis); it is lacking a few other important autoimmune diseases which are frequently associated with vitiligo such as autoimmune thyroid disease, adult-onset type 1 diabetes mellitus, pernicious anemia, systemic lupus erythematosus, and Addison disease. ^1^^,^^2^
2. Further, in the study, it is not clear which type of Vitiligo has been taken into account. It would be interesting and rather more significant if authors could integrate the different types of Vitiligo into consideration. Since the disease pathology varies between different types of Vitiligo as well as disease activity can further be considered in such DEG and gene interaction studies.
3. The demographic data of such patients can be linked along with the details of the type of disease, disease onset, duration of disease, VASI score, disease activity, etc. These data would be more meaningful with respect to the treatment aspect as well and can be very well correlated with the gene expression data in Vitiligo as well as associated autoimmune diseases.
4. Another important aspect to confirm in the present study is that: Did the data used by the authors for the differentially expressed genes in each disease were validated with respect to medication? Importantly, whether the expression data obtained from the patients differentiate who were on treatment or without treatment? Because treatment would surely affect the gene expression in these patients. Hence, it would be good if the authors could segregate and take the data accordingly.
5. Authors must avoid the term ‘auto-inflammatory disease’ for Vitiligo. Vitiligo is an autoimmune disease. The autoinflammatory theory mentioned here is not the correct term to use and it must be ‘autoimmune theory’. [The autoinflammatory theory is perhaps the most widely accepted theory that discusses the occurrence of Vitiligo.]

**Technical and utility concerns:**

1. The software should have features to generate clustered heat maps for the selected DEGs in vitiligo, psoriasis, RA and MS.
2. The software should also provide an option to compare DEG in lesional skin vs non-lesional skin of vitiligo patients.
3. Similarly, the software should provide an option to compare DEG within different types of vitiligo, for example, generalized vitiligo vs localized vitiligo.
4. The software should provide features to generate boxplots for the expression values for genes comparing expression between vitiligo (lessional, non-lessionals) and the different disease conditions (vitiligo, psoriasis, RA, MS).
5. It would be good to add features in the software to search the levels of a particular gene from the database.
6. The software should add the data of DEGs within particular cells/tissue and features to compare the DEG within different cell types/tissues.
7. The software should provide an option to carry out a meta-analysis of a particular gene in all the disease conditions (vitiligo, psoriasis, RA, MS), which would be beneficial to understand their role in the cross talk between the different disease conditions (vitiligo, psoriasis, RA, MS).
8. The software should have features to link the DEG to their respective signaling pathways, which will be crucial to pinpoint the role of the altered gene expression in these conditions.
9. The software should have an option to compare DEG of the available genes for drug treated group vs non-treated group.
10. The authors should provide video tutorials for the different features of the software, which will be helpful for first time users to easily navigate through the user interface.
11. Figures in the article could be provided with good resolution. The labeling and annotation in the figures are not visualized clearly (especially in Fig. 1, 2, 4 & 5C).

Are the conclusions about the tool and its performance adequately supported by the findings presented in the article?

Yes

Is the rationale for developing the new software tool clearly explained?

Yes

Is the description of the software tool technically sound?

Yes

Are sufficient details of the code, methods and analysis (if applicable) provided to allow replication of the software development and its use by others?

Partly

Is sufficient information provided to allow interpretation of the expected output datasets and any results generated using the tool?

Partly

Reviewer Expertise:

Immunogenetics, disease biology, vitiligo, autoimmunity

We confirm that we have read this submission and believe that we have an appropriate level of expertise to confirm that it is of an acceptable scientific standard, however we have significant reservations, as outlined above.

## References

[1] : Epidemiology of vitiligo and associated autoimmune diseases in Caucasian probands and their families. *Pigment Cell Res*.2003;16(3) : 10.1034/j.1600-0749.2003.00032.x 208-14 10.1034/j.1600-0749.2003.00032.x 12753387

[2] : Early disease onset and increased risk of other autoimmune diseases in familial generalized vitiligo. *Pigment Cell Res*.2005;18(4) : 10.1111/j.1600-0749.2005.00242.x 300-5 10.1111/j.1600-0749.2005.00242.x 16029422

# Peer review: Reviewer response for version 1

**DOI:** 10.5256/f1000research.28377.r70478

**Published:** 2020-09-21

**Authors:** Dinesh Gupta

**Affiliations:** 1 Translational Bioinformatics Group, International Centre for Genetic Engineering and Biotechnology (ICGEB), New Delhi, Delhi, India

VIRdb2.0 is an integrative database for vitiligo research, integrating it's links to Psoriasis, Multiple Sclerosis, Rheumatoid Arthritis, and Diabetes. The database release 2.0 is developed with additional features over the previous version.

 Summarily, the database is a collection of differentially co-expressed genes involved in the crosstalks with Vitiligo and associated autoimmune disorders. This looks like a good resource for researchers interested in Vitiligo research. Few concerns and suggestions, which the authors should address:

1. The manuscript should illustrate a few specific examples to demonstrate the utility of the database, apart from showing the overall capabilities.
2. The database site should include a tutorial for users, demonstrating the database tools and searches.
3. A new page to be created to highlight "what's new in this release".
4. It will be nice if the authors could include gene regulatory networks too, involving the role of common TFs in the related diseases.
5. It will be nice if some data on the druggability of a few common hubs could be highlighted too.
6. Few logical search options should be considered to make the database search more useful.

Are the conclusions about the tool and its performance adequately supported by the findings presented in the article?

Yes

Is the rationale for developing the new software tool clearly explained?

Yes

Is the description of the software tool technically sound?

Yes

Are sufficient details of the code, methods and analysis (if applicable) provided to allow replication of the software development and its use by others?

Partly

Is sufficient information provided to allow interpretation of the expected output datasets and any results generated using the tool?

Partly

Reviewer Expertise:

Translational Bioinformatics, Systems biology, database, and algorithm development.

I confirm that I have read this submission and believe that I have an appropriate level of expertise to confirm that it is of an acceptable scientific standard, however I have significant reservations, as outlined above.

# Response

**Published:** 2021-01-27

**Authors:** Abhishek Sengupta

**Affiliations:** Amity University, India

Dear Sir,

 We thank you for the generous and concise comments on our manuscript. We have incorporated the suggestions and changes have been added to the manuscript text also.

 Q.1 The manuscript should illustrate a few specific examples to demonstrate the utility of the database, apart from showing the overall capabilities.

**As per the suggestion, we have incorporated a case study at the Home page of the database, which demonstrates the use of the database to construct an interaction network for the intersecting genes between Rheumatoid Arthritis and Vitiligo and further use Cytoscape tool to import and analyse the network using mCODE app as a demonstration of the utility of the database.**

 Q.2 The database site should include a tutorial for users, demonstrating the database tools and searches.

**A short tutorial has been incorporated at the Home page highlighting the sections and the process of retrieval from the sections. Complete code for accessing the database is already available at GitHub.**

 Q.3 A new page to be created to highlight "what's new in this release".

**A new page has been created to highlight "what's new in this release" as advised.**

 Q.4 It will be nice if the authors could include gene regulatory networks too, involving the role of common TFs in the related diseases.

**A network gallery is available under the “Browse Section”. The visualization of the network of the intersecting genes with D3-Fore is available. These networks have been generated using GeneMania plugin from Cytoscape. Further, the analysis was also done to elaborate the Gene Ontology for the intersecting DEGs, the data is available in Appendix-VI. Further, we aim to build more towards the on this in our future studies.**

 Q.5 It will be nice if some data on the druggability of a few common hubs could be highlighted too.

**We have incorporated ADMET studies for the compounds that we have used for the purpose of screening and the excel files are also available in the “Downloads Section”**

 Q.6 Few logical search options should be considered to make the database search more useful.

**Since, this is a static database, for now, we will incorporate the logical search options in our next version of the database/tool.**
